# Importance of saline contrast transthoracic echocardiography for evaluating large right-to-left shunt in patent foramen ovale associated with cryptogenic stroke

**DOI:** 10.1007/s10554-021-02418-6

**Published:** 2021-09-21

**Authors:** Yoichi Takaya, Rie Nakayama, Teiji Akagi, Fumi Yokohama, Takashi Miki, Koji Nakagawa, Norihisa Toh, Hiroshi Ito

**Affiliations:** Department of Cardiovascular Medicine, Dentistry and Pharmaceutical Sciences, Okayama University Graduate School of Medicine, 2-5-1 Shikata-cho, Kita-ku, 700-8558 Okayama, Japan

**Keywords:** Patent foramen ovale, Right-to-left shunt, Cryptogenic stroke, Transthoracic echocardiography

## Abstract

Transcatheter closure of patent foramen ovale (PFO) is an effective therapy for preventing recurrent stroke in very specific patient cohorts, such as cryptogenic stroke (CS). The identification of high-risk PFO, which is more likely to be linked to CS, is essential. This study aimed to assess the accuracy of saline contrast transthoracic echocardiography (TTE) for evaluating large right-to-left (RL) shunt. We enrolled 119 patients with or without CS who were confirmed to have PFO by transesophageal echocardiography (TEE) or catheterization. The severity of RL shunt evaluated by TTE and TEE was classified as follows: small (< 10 microbubbles), moderate (10–20 microbubbles), and large (> 20 microbubbles). With TTE, large RL shunt was observed in 94 (79%) of 119 patients, including 66 of 74 with CS and 28 of 45 without CS. With TEE, large RL shunt was observed in 33 (28 %) patients, including 26 with CS and 7 without CS. TTE showed large RL shunt more frequently than TEE (p < 0.01). Large RL shunt evaluated by TTE had a sensitivity of 89 % and an accuracy of 70 % for the association with CS, whereas large RL shunt evaluated by TEE had a sensitivity of 35% and an accuracy of 56 %. Accuracy was significantly greater in TTE than in TEE (p = 0.02). In conclusion, TTE identified large RL shunt associated with CS with higher sensitivity and accuracy compared to TEE. Our findings suggest that the decision for device closure should be made based on the severity of RL shunt by TTE.

## Introduction

Patent foramen ovale (PFO) is linked with various diseases, including cryptogenic stroke (CS) [1–6]. Since randomized trials have demonstrated that transcatheter closure of PFO reduces the recurrence of stroke at higher rates compared to medical therapy [7–9], the relationship between PFO and CS has become increasing interest. The prevalence of PFO is approximately 25 % in the general population [10]. Thus, the diagnosis of high-risk PFO, which is more likely to be linked to CS, is important.

Large right-to-left (RL) shunt is effective to stratify PFO for an increased risk of CS. Saline contrast transesophageal echocardiography (TEE) remains the standard reference for assessing the severity of RL shunt [11, 12]. The RESPECT and REDUCE trials enrolled patients on the basis of TEE assessments [7, 8]. However, TEE often has the difficulty in accurately evaluating large RL shunt due to an insufficient Valsalva maneuver. A lower right atrial pressure caused by the fasting state of TEE leads to a reduction in the right and left atrial pressure gradient. Whereas, saline contrast transthoracic echocardiography (TTE) has been reported to be useful for PFO detection with a high sensitivity and specificity [13]. TTE does not require the fasting state and can obtain an adequate Valsalva maneuver. TTE may identify more large RL shunt as compared to TEE given the differences in loading conditions, such as Valsalva maneuver and hemodynamics. Therefore, TTE may be a better imaging modality for decision making with regard to device closure. This study aimed to assess the accuracy of TTE compared to TEE for evaluating large RL shunt associated with CS.

## Methods

### Study population

A total of 119 constructive patients with or without CS who were confirmed to have PFO in our institution from June 2015 to November 2020 were enrolled retrospectively. Presence of PFO was confirmed by TEE or cardiac catheterization. Patients with CS were proven to have cerebral infarction by magnetic resonance imaging. CS was diagnosed by a neurologist based on the exclusion of all other identifiable causes of stroke, such as large artery atherosclerosis, cardioembolism, small vessel disease, and arterial dissection, using brain and carotid imaging, electrocardiography, and echocardiography. This study classified patients with migraine as the group of patients without CS. They were planned for transcatheter PFO closure to treat migraine. All patients provided written informed consent for examination. The study was approved by the ethics committee of our institution.

## Saline contrast TTE

TTE using a 2.5- to 3.5-MHz probe with harmonic imaging (iE33 with an S5-1 probe; Philips Medical Systems, Best, The Netherlands, and Artida with a PST-25BT probe; Canon Medical Systems, Otawara, Japan) was performed. TTE images were obtained in an apical four-chamber view or a subcostal four-chamber view. Gain settings were individually adjusted to optimize visualization of the interatrial septum and agitated saline contrast. Saline contrast was produced by 1 mL air, 1 mL blood, and 8 mL saline, and was agitated between two 10 mL syringes connected with a three-way stopcock. The Valsalva maneuver was performed when the patient inhaled. TTE with the spontaneous Valsalva maneuver was performed at two times. Agitated saline contrast was injected from an antecubital vein immediately after applying the Valsalva maneuver, and the Valsalva maneuver was released at opacification of the right ventricle. If the spontaneous Valsalva maneuver was inadequate, the epigastric compression was added during the Valsalva maneuver [14]. TTE images were digitally stored. The maximum number of microbubbles appearing in the left ventricle was counted in a single frame. The severity of RL shunt was classified into three groups by independent cardiologists who were unaware of the status of the patient. Large RL shunt was defined as > 20 microbubbles, moderate RL shunt was defined as 10–20 microbubbles, and small RL shunt was defined as < 10 microbubbles.

## Saline contrast TEE

TEE using a 5-MHz multiplane probe (iE 33 with an X7-2t probe; Philips Medical Systems) was performed under local anesthesia. If needed, intravenous sedation was administered at the time of probe insertion. The severity of RL shunt was evaluated at the end of the routine examination when a patient was less sedated to enable performance of the spontaneous Valsalva maneuver. First, agitated saline contrast was injected at rest to assess the potential of pulmonary arteriovenous malformations. Second, agitated saline contrast was injected immediately after applying the Valsalva maneuver, and the Valsalva maneuver was released at opacification of the right atrium. The Valsalva maneuver was considered effective if leftward bulging of the interatrial septum was observed. Saline contrast injection was repeated at two times. The maximum number of microbubbles appearing in the left atrium was counted in a single frame. Similar to TTE, the severity of RL shunt was classified into three groups: large RL shunt (> 20 microbubbles), moderate RL shunt (10–20 microbubbles), and small RL shunt (< 10 microbubbles). The anatomical characteristics, such as the height of PFO, the length of PFO tunnel, and the presence of atrial septal aneurysm, were evaluated.

## Variability

Inter- and intra-observer differences were analyzed in all TTE and TEE images. The severity of RL shunt was evaluated by two blinded observers and by a single observer at two different times. Percentage agreement was used to assess the inter- and intra-observer variabilities.

### Statistical analysis

Data are presented as mean ± standard deviation for continuous variables and as number and percentage for categorical variables. Differences between two groups were analyzed by the *t* test for continuous variables and the χ^*2*^ test for categorical variables. The sensitivity, specificity, and accuracy of large RL shunt of TTE and TEE for the association with CS were analyzed. Accuracy was defined as (true positive) + (true negative) / total in sample. Comparisons were analyzed using the Fisher’s exact test. Statistical analysis was performed using JMP version 14.0 (SAS Institute Inc., Cary, NC, USA), and significance was defined as p < 0.05.

## Results

### Patient characteristics

The mean age of patients was 48 ± 15 years, and 63 patients were male. Seventy-four patients had CS, and 45 patients did not have CS. There were no patients who had non-cryptogenic stroke. Pulmonary arteriovenous malformations were not observed by TEE.

Clinical characteristics between patients with CS and those without CS are shown in Table 1. Patients with CS were older than those without CS. The heigh of PFO and the length of PFO tunnel were greater in patients with CS than in those without CS. Patients with CS more frequently had atrial septal aneurysm compared to those without CS.

**Table 1:** Clinical characteristics

	Patients with CS (n = 74)	Patients without CS (n = 45)	p
Age, years	53 ± 13	38 ± 15	< 0.01
Male	44 (59%)	19 (42%)	0.07
Hypertension	16 (22%)	5 (11 %)	0.15
Dyslipidemia	12 (16%)	5 (11%)	0.44
Diabetes mellitus	3 (4%)	1 (2%)	0.59
Smoking	16 (22%)	8 (18%)	0.62
PFO characteristics			
Height	2.3 ± 1.2	1.6 ± 0.9	< 0.01
Tunnel length	8.8 ± 3.7	7.0 ± 3.6	< 0.01
Atrial septal aneurysm	37 (50%)	5 (11%)	< 0.01

*CS* cryptogenic stroke, *PFO* patent foramen ovale

## Large RL shunt

**Fig. 1:**
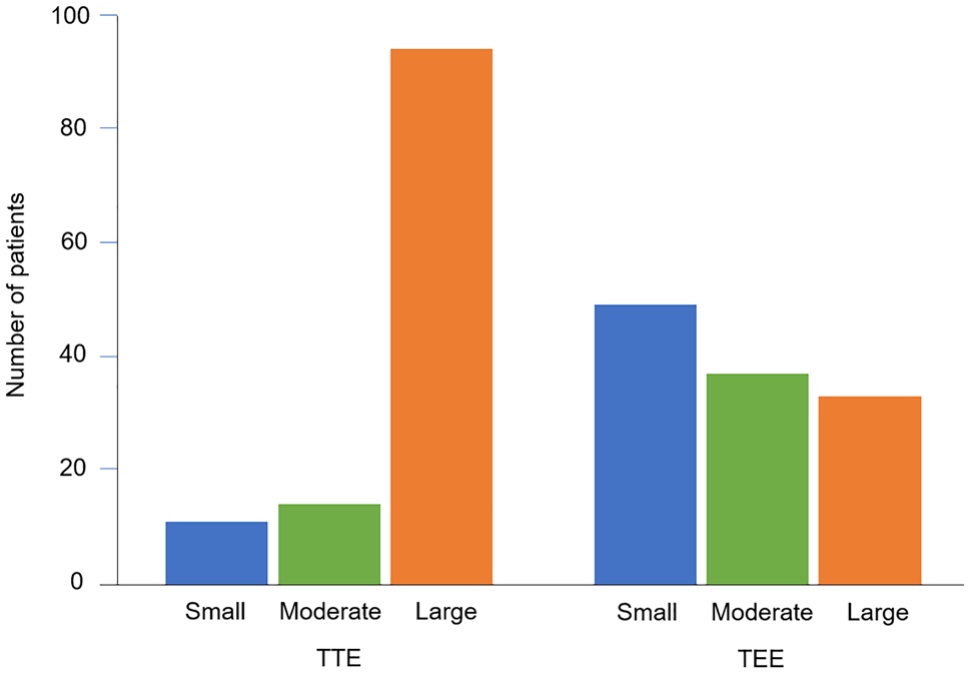
RL shunt evaluated by saline contrast TTE and TEE. TTE shows large RL shunt more frequently than TEE

With saline contrast TTE, large RL shunt was observed in 94 (79 %) of 119 patients, including 66 of 74 patients with CS and 28 of 45 patients without CS, moderate RL shunt was observed in 14 (12 %) patients, and small RL shunt was observed in 11 (9%) patients. With saline contrast TEE, large RL shunt was observed in 33 (28 %) of 119 patients, including 26 of 74 patients with CS and 7 of 45 patients without CS, moderate RL shunt was observed in 37 (31 %) patients, and small RL shunt was observed in 49 (41 %) patients (Fig. 1). TTE showed large RL shunt more frequently than TEE (p < 0.01).

**Fig. 2:**
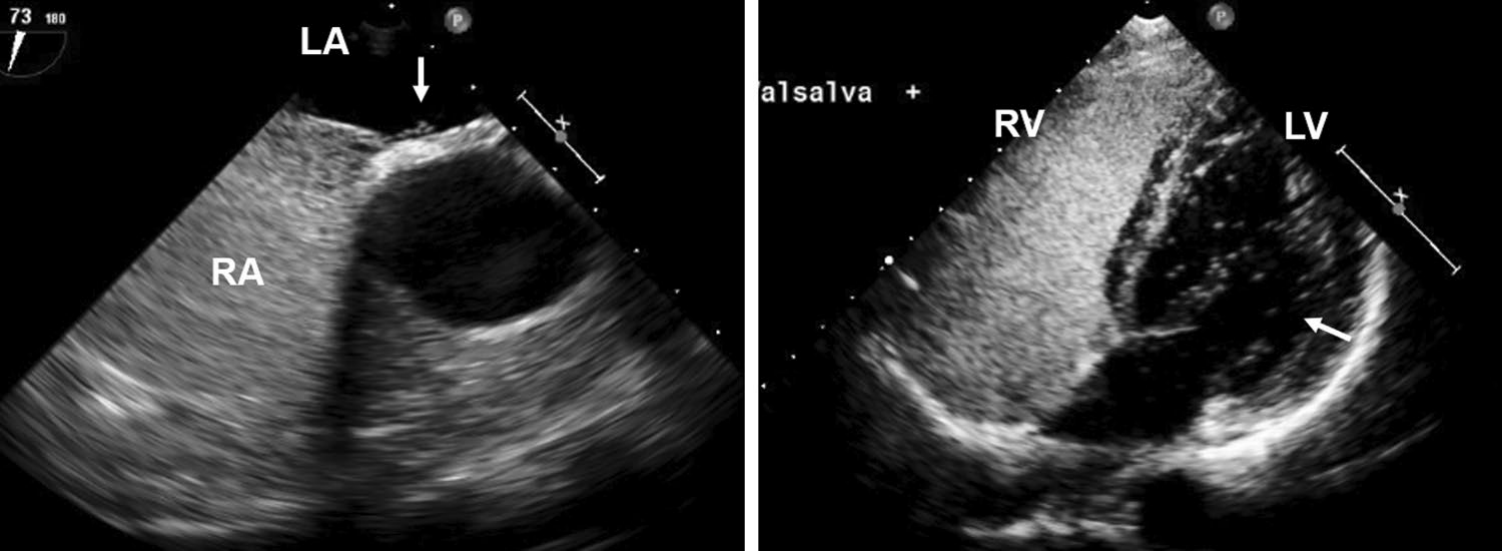
Representative case. Saline contrast TEE (left) shows small RL shunt with a few microbubbles appearing (arrow) in the left atrium, whereas saline contrast TTE (right) shows large RL shunt with > 20 microbubbles appearing (arrow) in the left ventricle. *LA* left atrium, *LV* left ventricle, *RA* right atrium, *RV* right ventricle

Differences in the severity of RL shunt between saline contrast TTE and TEE are shown in Table 2. TTE showing a greater severity of RL shunt than TEE was observed in 74 (62 %) patients, including 34 patients with large RL shunt on TTE and moderate RL shunt on TEE, 29 patients with large RL shunt on TTE and small RL shunt on TEE, and 11 patients with moderate RL shunt on TTE and small RL shunt on TEE. The same severity of RL shunt between TTE and TEE was observed in 42 (35 %) patients. TTE showing a less severity of RL shunt than TEE was observed in 3 (3 %) patients. Figure 2 shows a representative case demonstrating the difference in the severity of RL shunt between saline contrast TTE and TEE. Only a few microbubbles appeared in the left atrium with TEE, but large RL shunt (> 20 microbubbles) was observed with TTE.

**Table 2:** Severity of RL shunt between saline contrast TTE and TEE

TTE	TEE			
	Small	Moderate	Large	Total
Small	9 (8%)	1 (1%)	1 (1%)	11 (9%)
Moderate	11 (9%)	2 (2%)	1 (1%)	14 (12%)
Large	29 (24%)	34 (29%)	31 (26%)	94 (79%)
Total	49 (41%)	37 (31%)	33 (28%)	119 (100%)

*RL* right-to-left, *TEE* transesophageal echocardiography, *TTE* transthoracic echocardiography

The sensitivity, specificity, and accuracy of large RL shunt of saline contrast TTE and TEE for the associated with CS are shown in Table 3. Large RL shunt evaluated by TTE had a sensitivity of 89%, a specificity of 38 %, and an accuracy of 70%. Large RL shunt evaluated by TEE had a sensitivity of 35 %, a specificity of 84%, and an accuracy of 54%. The sensitivity and the accuracy of large RL shunt of TTE were significantly greater than those of TEE (p < 0.01, p = 0.02). The specificity of large RL shunt of TEE was greater than that of TTE (p < 0.01).

**Table 3:** Large RL shunt of saline contrast TTE and TEE for the association with CS

	Sensitivity	Specificity	Positive predictive value	Negative predictive value	Accuracy
TTE large RL shunt	89%	38%	70%	68%	70%
TEE large RL shunt	35%	84%	79%	44%	54%

*CS* cryptogenic stroke, *RL* right-to-left, *TEE* transesophageal echocardiography, *TTE* transthoracic echocardiography

## Reproducibility

There were 99 % agreement in the classification of RL shunt of TTE and TEE by two blinded observers, and 98 % agreement by a single observer assessing twice.

## Discussion

The major findings of the present study were: (1) saline contrast TTE showed large RL shunt more frequently than TEE, and (2) large RL shunt evaluated by TTE had greater sensitivity and accuracy for the association with CS compared to that evaluated by TEE, and large RL shunt evaluated by TEE had greater specificity compared to that evaluated by TTE. To the best of our knowledge, this is the first study to show the efficacy of TTE for evaluating large RL shunt associated with CS.

Since randomized trials, such as the RESPECT, REDUCE, and CLOSE trials, have demonstrated the benefits of transcatheter closure for the reduction of stroke [7–9], transcatheter closure of PFO is expected to increase as a therapeutic option. PFO is common, but not all PFOs are the same. It is necessary to diagnose high-risk PFO, which is more likely to be linked to CS. Larger RL shunt carries a greater potential for transseptal passage of thrombus. Large RL shunt is a particularly important risk factor for an increased likelihood of CS [15–17]. Furthermore, the RESPECT and REDUCE trials revealed that the effect of transcatheter PFO closure for preventing CS was increased in patients with large RL shunt evaluated by TEE [7, 8]. The CLOSE trial included patients with atrial septal aneurysm or large RL shunt evaluated by TEE or TTE, and demonstrated that transcatheter closure was superior, with a lower rate of stroke (in fact, no stroke at all), compared to medical therapy [9]. With the advent of transcatheter closure of PFO, patient selection has become important. Large RL shunt is useful to identify patients who obtain greater benefit from transcatheter closure. Therefore, accurate assessment of large RL shunt is essential.

Saline contrast TEE is used for the assessment of RL shunt. The amount of RL shunt evaluated by TEE has reportedly been associated with CS [15, 16], however several studies have shown that the distribution of RL shunt is not linked to CS [11, 18, 19]. These conflicting results could be explained by differences in the performance of the Valsalva maneuver. The Valsalva maneuver increases intrathoracic pressure and decreases venous return and left atrial pressure during the strain phase. Rebound venous return after the release increases right atrial pressure and the gradient between right and left atrial pressures, leading to opening of the flap-like foramen ovale and facilitating RL shunt. Thus, an adequate Valsalva maneuver is essential to assess the severity of RL shunt in patients with PFO.

Saline contrast TEE has technical limitations in the accurate assessment of RL shunt. The Valsalva maneuver is insufficient due to probe insertion. Under sedation, the Valsalva maneuver itself is difficult to perform. The fasting state causes a lower right atrial pressure, leading to a reduction in the right and left atrial pressure gradient with the Valsalva maneuver. Thus, TEE could result in an underestimation of the severity of RL shunt [20]. Whereas, saline contrast TTE has increasingly been utilized for the detection of PFO. The diagnosis of PFO has greatly improved with high sensitivity and specificity [13, 14]. TTE has an advantage in that patients are able to adequately perform the Valsalva maneuver. On the basis of these findings, TTE can be reliable for evaluating RL shunt. Additionally, TTE is simple to use, easily available, and low cost [21–23].

This study has the impact on changing clinical practice. In general, patients with CS will receive TTE assessments first. Thereafter, they would undergo TEE for anatomic confirmation. Our findings suggest that the decision for device closure should be made based on the severity of RL shunt by TTE rather than TEE. TTE can be a better imaging modality for decision making in patients with CS.

Nevertheless, TEE also play an important role in clinical practice in patients with CS. TEE is still recommended if no RL shunt identified on TTE and high clinical suspicion remains. TEE is required to assess morphological characteristics of PFO for planning device closure, including the discrimination between PFO and atrial septal defect. TEE is useful for excluding pulmonary arteriovenous malformations, evaluating left atrial appendage thrombosis, and searching for other comorbidities. Thus, both TTE and TEE are necessary for the management in patients with CS.

The present study had some limitations. First, the number of patients was small. Second, the severity of RL shunt depended on the degree of Valsalva maneuver. However, the assessment of RL shunt was uniform because TTE and TEE with the Valsalva maneuver were performed at a single institution. Third, the Valsalva maneuver on TEE was performed when sedation weakened in this study. It may be possible to perform TEE only with pharyngeal anesthesia in order to apply complete Valsalva maneuver. Finally, the efficacy of large RL shunt of TTE was not assessed in an independent population. The accuracy in this study represented a best-case scenario.

In conclusion, saline contrast TTE can identify large RL shunt more frequently than TEE. Large RL shunt evaluated by TTE provides greater sensitivity and accuracy for the association with CS. TTE can be valuable for evaluating large RL shunt associated with CS, and may be effective for stratifying patients who should undergo transcatheter PFO closure.

## Data Availability

This study is based on data from our institution. The data are available from the corresponding author on reasonable request.

## Code availability

Not required.
